# Traditional Approaches for Company Valuation Are Flawed for Valuing *In Vivo* Gene Therapy Companies

**DOI:** 10.1089/humc.2018.29037.gam

**Published:** 2018-12-17

**Authors:** Gbolahan Amusa, Taylor Feehley, J. Kipchirchir Bitok, Geulah Livshits, Natalya Gertsik

**Affiliations:** Equity Research Department, Chardan, New York, New York.

**Keywords:** gene therapy, company valuation, stock prices, shared value, sustainability

## Abstract

The era of gene therapy has begun. In recent years, potentially breakthrough datasets and rapidly expanding company pipelines have begun to overshadow the unfulfilled promise characteristic of the gene therapy sector in decades prior. One barometer for progress in the space can be seen in stock markets, where NASDAQ-listed *in vivo* gene therapy companies we follow have increased from 4 companies with $1.9 billion in market capitalization on January 31, 2014, to 24 companies with $30.5 billion in market capitalization on October 31, 2018. For many in the financial community, a tangible signal for the emergence of the broader gene therapy space is the recent notable mergers and acquisitions activity, a signal that previously heralded the arrival of blockbuster biotechnologies like monoclonal antibodies. Notably, Novartis' $8.7 billion acquisition of *in vivo* adeno-associated virus 9–based gene therapy player, AveXis, earlier this year has focused many on looking for new investment opportunities in the space, thereby increasing interest in the valuation of gene therapy companies. This perspective discusses the theoretical underpinnings of company valuation and explains why traditional approaches have limitations when valuing *in vivo* gene therapy companies, which produce single treatments that may achieve durable or curative benefits. We use the AveXis case study to illustrate certain points on the valuation of breakthrough innovation that we think have broader applicability throughout the *in vivo* gene therapy space. This publication is the first in a three-part series. Future discussions in this series on *in vivo* gene therapy companies will explore real-world approaches and considerations that have already proven successful in mitigating the limitations of traditional valuation approaches as well as those that may soon emerge.

## Introduction

The U.S. Food and Drug Administration (FDA) defines gene therapy products as “all products that mediate their effects by transcription or translation of transferred genetic material [whether DNA or RNA], or by specifically altering host (human) genetic sequences.”^1^ For our definition, we include only those products used to introduce exogenous genetic material into cells to treat or prevent a disease. Since *in vivo* gene therapies are thus far associated with both one-time treatments and one-time payments, such therapies create unique company valuation issues described in this perspective. The scope of this perspective is thus limited to *in vivo* gene therapy companies.

For decades, many hoped that *in vivo* gene therapies would be a solution for a variety of human diseases. In more recent years, potentially breakthrough results were finally demonstrated with *in vivo* gene therapies in neuromuscular and muscular diseases, hemophilia, immunodeficiencies, and inherited retinal diseases. For many, the exemplification of the promise of *in vivo* gene therapies being fulfilled is the breakthrough result shown so far for AveXis' AVXS-101 (ZOLGENSMA, onasemnogene abeparvovec), which may have proven transformative in rescuing children from a leading cause of infant mortality, spinal muscular atrophy type 1.^2^ As gene therapies continue to move from academia to industry, companies are tackling the complexity of gene therapy technologies by using the tools of the genomics and precision medicine revolution to better design and target products, while using new vector and transgene technologies to limit historic issues on immunogenicity.^3^

To keep pace with innovation, the FDA has devoted additional resources to gene therapies, cleared its backlog of orphan drug designations, focused on genetic rather than phenotypic diagnoses (*e.g.*, *RPE65* mutations for treatment with voretigene neparvovec^4^), issued disease-specific gene therapy guidance documents,^5^ and permitted certain modifications of gene therapy products during clinical development (*e.g*., changing the transgene from AMT-060 to AMT-061^6^). Further, the FDA is considering accelerated approvals based on convincing phase 1/2 datasets and is allowing companies to demonstrate durability after products are on the market. The stated view of FDA Commissioner Gottlieb is instructive: “Over the next several years, we'll see [gene therapy] become a mainstay of treating, and probably curing, a lot of our most devastating and intractable illness.”^7^ Indeed, 2017 saw a tangible regulatory signal for the arrival of gene therapies, namely the first three FDA approvals of gene therapies—two *ex vivo* gene-modified cell therapies for cancer (tisagenlecleucel and axicabtagene ciloleucel) and an *in vivo* gene therapy (voretigene neparvovec).

**Figure 1.**
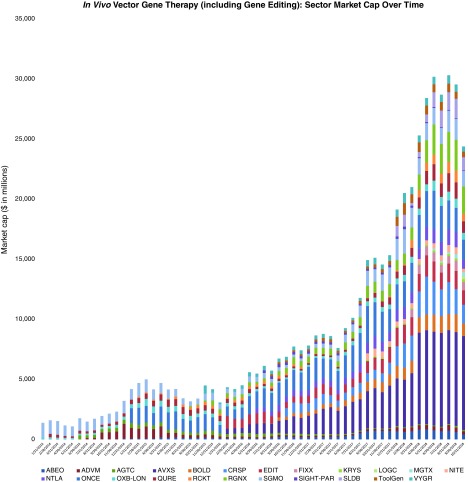
The *in vivo* gene therapy space (including gene editing)—market cap progression from end-January 2014 to end-October 2018.

Perhaps as notable as the first regulatory approvals for gene therapies has been the series of announced acquisitions of gene therapy companies. On August 28, 2017, Gilead Sciences announced its acquisition of *ex vivo* gene-modified cell therapy company, Kite Pharma, for approximately $12 billion,^8^ and on January 22, 2018, Celgene Corporation announced its plans to acquire Kite's peer, Juno Therapeutics, for approximately $9 billion.^9^ Just months later, on April 9, 2018, Novartis announced its agreement to acquire *in vivo* AAV9-based gene therapy company, AveXis, for $8.7 billion.^10^ This interest in obtaining gene therapy capabilities was not limited to bigger biopharmaceutical companies like Gilead, Celgene, and Novartis, since smaller companies targeting genetic diseases also began to transition from developing traditional therapeutics to also developing gene therapies via acquisitions of companies or technologies. Examples include: Amicus Therapeutics, Ophthotech Corporation, PTC Therapeutics, Sarepta Therapeutics, and Ultragenyx Pharmaceutical. For some observers, the confluence of positives for the gene therapy space justifies the vast expansion in market capitalization for *in vivo* gene therapy companies we follow (Fig. 1).

## The Value of Gene Therapies: a Societal Perspective

**Figure 2.**
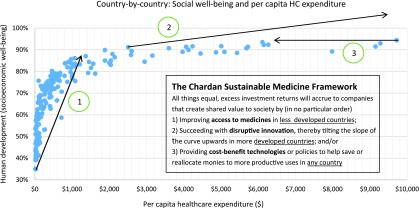
Higher healthcare consumption is associated with higher societal measures of well-being (*i.e.*, real value for society).^19^ HC, healthcare.

The World Health Organization established healthcare as a causal factor for economic progress^11^ and has been an advocate for and instrumental in the global healthcare insurance and infrastructure expansion seen in the past 20 years in countries like Brazil, China, Ghana, and Thailand.^12^ The ongoing move of healthcare systems toward cost-effectiveness considerations to deal with the high cost but marginal value of many medicines is increasing the importance of real value being delivered by therapeutics. Based on the Chardan Sustainable Medicine Framework (CSMF) shown in Fig. 2, companies that deliver the most value to global society (via disruptive innovation, cost savings, or access to medicine initiatives) can be expected to have higher company valuations from sharing in the value delivered. Indeed, gene therapy companies that succeed will not only see higher potential for product uptake from transforming clinical practice (CSMF dimension 2), but also lower potential for onerous government or private sector interventions, since gene therapies can save other healthcare costs, as has been shown with investigational hemophilia gene therapies^13^ (CSMF dimension 3). In the long term, gene therapies are among the best solutions for access to medicines initiatives in less-developed countries (CSMF dimension 1), since *in vivo* gene therapies are typically one-time treatments that may be delivered even where extensive healthcare infrastructure is missing. We remind readers that lower risk and higher reward is a potent mix of factors supporting higher company and/or sector valuations.

## The Basics of Company Valuation

Valuation work in the gene therapy space is routinely conducted by professionals in a number of fields, including investment banking, equity research, venture capital, and business or corporate development. The views of equity research (“sell side”) analysts may be the only ones more easily accessible through public forums; however, such views should be taken cautiously, given the long-established optimism bias that exists in sell-side research due to the conflicts of interest that can sometimes lead to fear of retaliation against analysts who issue Sell ratings on investment banking clients, and the tendency of analysts to err towards an optimistic consensus, or favor larger banking clients, rather than independence.^14^ These biases increase the importance of relying upon analysts known for research independence and intellectual honesty.

In the gene therapy space, company or asset valuation could be useful for a number of purposes, including

- Predicting the future stock price of a gene therapy company (*e.g*., after a proof-of-concept clinical trial readout),
- Predicting the valuation at which a gene therapy company could be sold to another biopharma company,
- Predicting the public market valuation for a private gene therapy company once it goes public, and
- Predicting the valuation for a private gene therapy company after a subsequent round of financing.

Various philosophies exist for valuing a company or an asset. Among the most widely accepted fundamental finance concepts is that the value of an asset should reflect the cash flows it generates. Less fundamental but oftentimes more lucrative approaches to company valuation include behavioral finance considerations, which involve valuing a company or an asset based on what others are or will be willing to pay for it. Depending on the goal at hand, an asset will be worth different amounts to different market participants.

**Figure 3.**
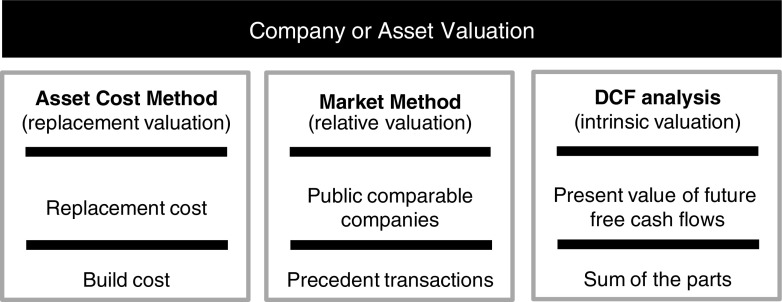
Company or asset valuation is often conducted using any of three methodologies: (1) an asset cost (or replacement) method, (2) a market (relative valuation) method, or (3) a discounted cash flow (DCF) (intrinsic value) analysis.

We consider three major approaches to company or asset valuation: (1) cost or replacement methods, (2) market or relative valuation methods, and (3) discounted cash flow or intrinsic value methods (see Fig. 3).

### The cost method

For the cost method, the idea is to value a company based on the cost to either replace or build its components. The ideal application of this method occurs when assets can be separated or treated separately and when there are active markets to price the separated assets. As such, this approach is rarely used for valuing publicly traded healthcare companies since, for example, important assets are often not feasible to build or replace due to intellectual property or other obstacles. Since there currently are no liquid markets for the critical components that make up a gene therapy company, this method cannot be applied routinely to the valuation of gene therapy companies.

### Market methods (relative valuation)

Most Wall Street equity valuations are relative valuations based on multiples derived from public comparable companies (trading comps) or precedent mergers and acquisitions transactions (deal comps).

An important advantage of relative valuation is that it is faster than intrinsic valuation, as relative valuation typically requires far fewer assumptions or calculations. For example, if the typical mergers and acquisitions transaction in a sector has occurred at a 50% premium to recent stock prices, one could argue that a 50% premium is justified for an acquisition being considered in the sector. The disadvantages are that relative valuation oftentimes leads to views consistent with consensus, which means that analysts relying on relative valuation methods in the *in vivo* gene therapy space would likely not have anticipated the vast outperformance of a company like AveXis. In addition, if a sector's multiples are inappropriately compressed, analysts will be more likely to derive inappropriately low company price targets based on using compressed comps. Such analysts are less likely to identify hidden value.

The steps in the application of relative valuation include

- Identify comparable companies (*e.g*., with similar cash flow, growth, risk, and technology profiles),
- Identify and calculate standardized metrics for valuation (*e.g*., a price-to-earnings ratio), and
- Apply the standardized metric to the company, making any necessary adjustments.

A limitation of using multiples from trading comps to value gene therapy companies is that gene therapy companies overwhelmingly lack revenues and have negative profitability. The limitations on using multiples from deal comps to value gene therapy companies include the limited universe of gene therapy company acquisitions, the wide dispersion of takeout valuations, and the difficulty in arguing that companies in precedent transactions are directly comparable to other companies in the space (*e.g*., some may feel AveXis is a unique story).

### Discounted cash flow analysis (intrinsic valuation)

Discounted cash flow (DCF) valuation is one of our most preferred tools for valuing *in vivo* gene therapy companies. DCF valuation is a fundamentally sound approach closely tied to finance theory. A DCF analysis for a gene therapy company, as with other companies, would require forecasting product revenues and associated expenses to derive cash flows, which are then discounted to a net present value (*i.e*., the value today of the company). The flexibility of DCF valuation gives it utility if, for example, a gene therapy analyst wants to understand the effects on valuation of any of the following scenarios:

- The initial revenues for a gene therapy will be higher due to upfront pricing on 10 years of assumed benefits,
- An expedited approval of the gene therapy is possible on breakthrough phase 1/2 datasets,
- Manufacturing costs may be higher initially due to gene therapy product complexity, or
- Promotion costs may be lower for gene therapies due to focused distribution (*e.g*., via key opinion leaders).

The advantages of a DCF analysis is that it can reduce the impact of the current market environment on valuation and can promote outside-of-consensus views (*e.g*., an early view that AveXis may vastly outperform). In addition, a DCF analysis can be applied when market multiples are not available when a new industry is emerging and/or when an industry is not yet profitable, as has been the case for the gene therapy space. The disadvantage of intrinsic valuation approaches is that such approaches are highly dependent on long-term forecasts and terminal value assumptions.

A limitation of the application of DCF analyses for *in vivo* gene therapy companies is the importance of the terminal value. The terminal value is the estimated value of a business beyond an explicit forecast period. For example, even if an analyst explicitly models gene therapy-derived cash flows as far as 2030, the terminal value (which assumes constant growth of cash flows beyond 2030) can still be 50–70% or more of a gene therapy company's valuation and thus provides analysts tremendous discretion, if the analyst chooses to use it, in valuing companies. In addition, DCF analyses are not typically adapted to value intangible assets such as brand value, which for example may have been a factor behind the 88% premium Novartis paid to acquire AveXis.

## Gene Therapy: Rapid Adoption With Upfront Pricing Limits the Use of Dcf Terminal Valuation

As above, terminal valuation (the present value of cash flows beyond the explicit forecast horizon) is typically 50–70% or more of DCF-derived company valuations. Here, we discuss how, assuming the same cash flows for a product over its lifetime, a scenario with front-end-loaded cash flows for a gene therapy (*e.g*., due to more rapid adoption in prevalent patients) may counterintuitively result in a lower DCF valuation due to the impact on terminal values. We believe this limitation in using DCF valuation for less diversified *in vivo* gene therapy companies (*e.g*., as was the case for AveXis) that will reinvest in growth can lead to lower valuations than is appropriate.

The issue on valuation arises because one-time administration of gene therapies challenges traditional models, since the potential benefits for gene therapies accrue for years after administration, but healthcare systems currently tie payments to delivery of therapeutics, as opposed to the benefits therapeutics provide. The challenges in the United States to establishing pay-for-performance (*i.e*., value-based or risk-sharing agreements to amortize the gene therapy price over time if durable benefits are shown) include Medicaid best-price regulations and federal anti-kickback statutes, which limit the ability of gene therapy companies to offer discounts or refunds for products losing efficacy as well as short (*e.g*., 2- to 3-year) tenures for Americans on health plans, which limit the willingness of health insurers to continue to pay for durable therapies if patients switch to new plans. For these reasons, pay-for-performance models may first emerge in Europe, where central payors can internalize both the benefits and costs of gene therapies.

An important upcoming precedent for the gene therapy space will be the price set for AVXS-101. In our March 28, 2016, AveXis research publication, we noted that “if a gene therapy product provides 10-year benefits that are in line with orphan drug Soliris, which is priced at >$400,000 per year, one could argue the gene therapy product should be priced at >$4 million upfront. … If instead, pay-for-performance models … are in place, it would be possible to price the drug at >$400,000 per year for each year of benefit.”^15^ Notably, AveXis' acquirer, Novartis, during its November 5, 2018, R&D Day, determined that the one-time price of treatment with AVXS-101 is “cost effective in the range of $4–$5 million.” The AVXS-101 price will be revealed at launch, potentially in 2019. We note draft scope documents for AVXS-101 already exist from cost-effectiveness organizations, National Institute for Health and Care Excellence^16^ and Institute for Clinical and Economic Review.^17^

**Figure 4.**
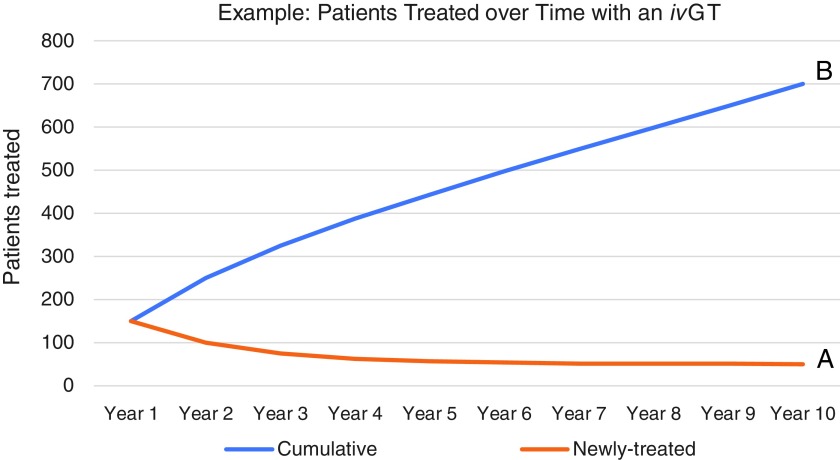
Patients treated with a disruptive *in vivo* gene therapy (*iv*GT)—Representation for the same gene therapy of **(A)** newly treated patients and **(B)** cumulatively treated patients.

**Figure 5.**
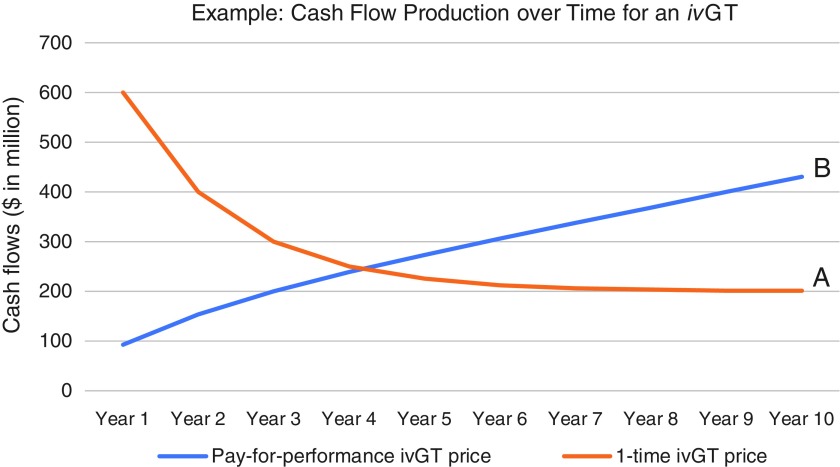
Payment systems—**(A)** one-time *in vivo* gene therapy (ivGT) prices for newly treated patients with a devastating disease like spinal muscular atrophy type 1 may be associated with declining cash flows over time, while **(B)** pay-for-performance price regimes, which reimburse for cumulatively treated patients, may be associated with increasing cash flows.

Consider that under a one-time pricing scheme for one-time *in vivo* gene therapies, particularly for a highly effective drug for a devastating disease, initial revenues and cash flows would come disproportionately from use in prevalent, or warehoused, patient populations. Once a high proportion of the prevalent population has been treated, then use in the incident population would contribute to a higher proportion of sales. Scenario A in Fig. 4 is an example of one such declining curve for newly treated patients per year, and scenario A in Fig. 5 is an example of the associated declining cash flows expected with a one-time pricing scheme. Alternatively, in Fig. 4, scenario B, under a pay-for-performance pricing scheme, the rising amount of cumulative patients treated represents the patients that would lead to payment, assuming durability of the gene therapy to at least 10 years; and scenario B in Fig. 5 represents the shape of the cash flow curve under a representative pay-for-performance model, where payment is provided for each year a patient sees a durable benefit from a gene therapy. This trajectory in scenario B is consistent with what is typically seen for a traditional therapeutic.

The two scenarios in Fig. 5 were chosen such that the areas under the curves (*i.e*., total cash flows generated over 10 years) are the same. Despite the same cash flows, the two scenarios would lead to drastic differences in company valuation under widely used methodologies. *Ceteris paribus*, valuation methodologies—such as the market (relative valuation) methods commonly used to value a profitable company like Novartis—which put more weight on forward financial estimates (*i.e*., from years 1 to 2) would value the company in scenario A of Fig. 5 much more highly than the one in scenario B, even though aggregate cash flows are the same. In contrast, valuation methodologies like a discounted cash flow (DCF) analysis—a more likely tool for yet-to-be profitable companies like AveXis—which put more weight on terminal financial estimates, would value the company in scenario A at a lower level than the one in scenario B. (Another way to think of this is the likely AveXis pattern of cash flows may have been worth more to Novartis investors than to AveXis investors, perhaps exposing a potential arbitrage and thus a driver for the acquisition.) We note that though scenario B (year 10 terminal cash flows of $431 million) would yield higher valuation in a DCF than scenario A (year 10 terminal cash flows of $201 million)*,* companies would prefer the cash flow profile in scenario A, since cash flows would be actualized sooner and could be reinvested in growth earlier (it takes 4 years to collect over half of the 10-year cash flows in scenario A vs. 7 years in scenario B), and therefore better ensure terminal growth beyond year 10. Traditional DCF valuation for potentially disruptive *in vivo* gene therapy companies like AveXis is therefore a limited tool that can lead to undervaluation.

## Gene Therapy Company Portfolio Scenarios and Implications for Valuation

**Figure 6.**
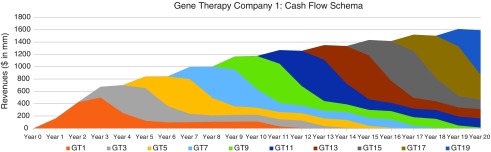
Well-diversified, highly productive gene therapy (GT) company cash flow schematic—assumes 10 regularly interspaced gene therapy launches every 2 years, over 20 years.

**Figure 7.**
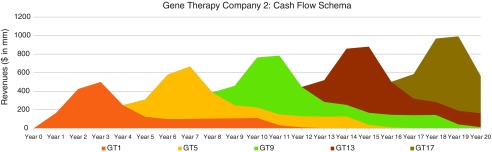
Moderately diversified, consistently productive GT company cash flow schematic—assumes five regularly interspaced gene therapy launches every 4 years, over 20 years.

**Figure 8.**
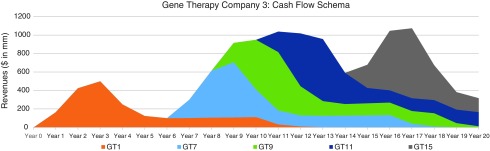
Moderately diversified, inconsistently productive GT company cash flow schematic—assumes 5 irregularly interspaced gene therapy launches, over 20 years.

In Figs. 6, 7, and 8, we consider three portfolio scenarios for gene therapy companies, with each scenario differing in (a) the number of gene therapy product launches in a company's portfolio, and (b) the timing of the gene therapy launches. The analysis shows the identified limitations in the use of DCFs to value gene therapy companies increases with (1) less product diversification, and/or (2) terminal valuations determined further after products peak.

Unlike the single-asset cash flow example seen in Fig. 5, we assume products reach peak revenues in year 3. Our assumptions:

- Gene therapy products target conditions without adequate treatment options.
- Peak cash flows of $500 million for each gene therapy are achieved in year 3 and subsequently adjusted for inflation.
- Launches occur as per the figure descriptions for Figs. 6, 7, and 8.

For the well-diversified, highly productive *in vivo* gene therapy company in Fig. 6, analyst choice of terminal year (*e.g*., year 17, year 18, year 19, or year 20) would have the least effect on the company's DCF valuation. For the moderately diversified, consistently productive *in vivo* gene therapy company in Fig. 7, terminal cash flows from year 17 to year 20 vary more. Finally, for the moderately diversified, inconsistently productive *in vivo* gene therapy company, we note year 17 terminal cash flows of $1,073 million, versus year 20 terminal cash flows of $315 million, leaving considerable room for analyst discretion in valuation, exposing a limitation in the use of DCF valuation.

## Conclusions, Future Perspectives, and the Reward for Creating Value for Society

Much of the promise of one-time administration *in vivo* gene therapies is the potential of such treatments to provide durable benefits or even cures for devastating diseases with high unmet medical needs. The advent of gene therapy has generated optimism that can be seen in the stock market performance of the broader sector; and, those adept at valuing the benefits offered by disruptive *in vivo* gene therapy companies have seen considerable rewards in recent years. For example, AveXis may have generated real value for society by transforming a leading genetic cause of infant mortality. The last trading price of AveXis (NASDAQ: AVXS), $217.83 on May 14, 2018, represents a 10.9× multiple (989.2% performance) versus the initial public offering price of $20.00 on February 11, 2016,^18^ and thus a tremendous reward to AveXis shareholders.

As the number of gene therapy companies continue to increase, and potentially transformative results continue to emerge, understanding the utility and limitations of applying traditional valuation methodologies to the space is increasing in importance. For example, the cash flows for disruptive *in vivo* gene therapies on the verge of entering the market may be valued more highly using market (relative valuation) methods than DCF (intrinsic valuation) approaches, due to rapid uptake and one-time, upfront pricing for years of durability. This phenomenon potentially explains the reason AveXis was acquired for much higher valuations than even bullish market views of AveXis anticipated. Moving forward, the most robust valuation methodologies over the long-term that are likely to predict share price performance should also focus on shared value (*i.e*., the value capture that gene therapies can achieve by first delivering real value to society).
